# Twists and turns: unraveling the mystery of ileosigmoidal knotting, a rare and intriguing acute intestinal obstruction (a report of 3 cases)

**DOI:** 10.11604/pamj.2024.49.92.42322

**Published:** 2024-11-26

**Authors:** Tariq Ahbala, Youssef Ibneloualid, Elhabib Lammat, Khalid Rabbani, Abdelouahed Louzi

**Affiliations:** 1 General Surgery, Mohammed VI University Hospital Center of Marrakech, Marrakech, Morocco

**Keywords:** Acute intestinal occlusion, ileosigmoid knotting, sigmoid colon, compound volvulus, case report

## Abstract

Among the rare and complex causes of acute intestinal occlusion: volvulus due to the ileosigmoid node. The latter is secondary to the terminal ileum which will wrap around the base of the sigmoid colon and its meso, resulting in the formation of a node. Its natural evolution is the ischemia of the ileum and sigmoid, necessitating prompt emergency surgical intervention as the primary treatment approach. In this series, we present three cases from the Department of Digestive Surgery at Arrazi Hospital, Mohammed VI University Hospital in Marrakech, while reviewing the physiopathology, the clinical presentation of this rare entity, as well as the different therapeutic options of the ileosigmoid nodes. We report three cases in the Department of Digestive Surgery at Arrazi Hospital, Mohammed VI University Hospital in Marrakech, in which the diagnosis of ileosigmoid knotting was made by means of computed tomography (CT) scan data and confirmed intraoperatively. Through these 3 reported cases and in the light of data from the literature, we will discuss the different diagnostic, therapeutic, and prognostic aspects of this very rare type of volvulus.

## Introduction

Intestinal knot syndromes represent rare yet life-threatening causes of closed double-loop obstructions. Among the various types described, ileosigmoid, ileoileal, appendico-ileal, ileocecal, and knotting by Meckel's diverticulum have been reported. Among them, ileosigmoid knotting is the most frequently documented variety in the literature. This condition involves the devitalization of both segments, carrying a grave prognosis unless urgent surgical intervention is performed.

The incidence of ileosigmoid nodes is significantly higher in developing countries and in patients of lower socioeconomic status [1-4]. In addition, it occurs most often in adult subjects over the age of forty, as well as males are affected more than females with a sex ratio of 1/4 being with an estimated percentage of 80.2% [5].

Due to its risk of rapid progression to ischemic necrosis of the ileum and sigmoid colon, causing acute generalized peritonitis and septic shock, the ileosigmoid node is associated with a very high mortality rate [5]. In order to prevent rapid deterioration leading to necrosis of the sigmoid colon and ileum, and thus death by septic shock, immediate surgical intervention is essential to save the patient.

## Patient and observation

### Case 1

**Patient information:** a 50-year-old farmer went to the emergency room for a complete stop of transit three days before admission, associated with abdominal pain. He had no medical or surgical history. At the time of examination, he had a tachycardia of 102 beats/min, blood pressure of 100/60 mmHg, and was apyretic at 37.2°C. Abdominal examination showed significant abdominal distension, diffuse tympanism, and absence of intestinal sounds with rectal touch and an empty rectal ampoule.

**Figure 1:**
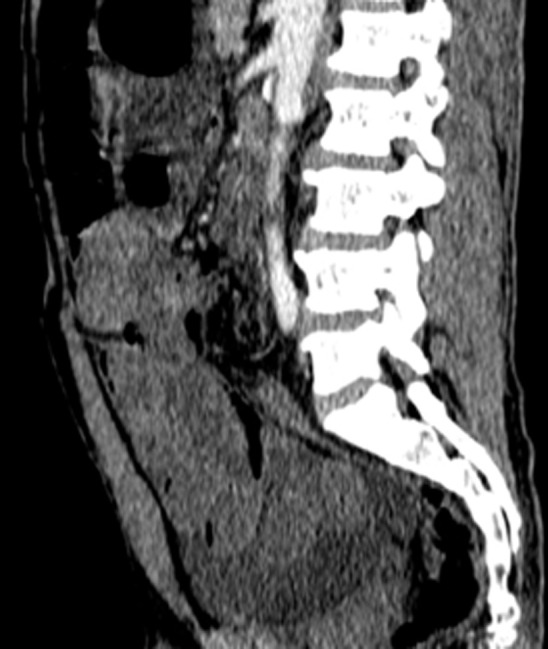
computed tomography in sagittal reconstructions, with injection of contrast product showing the distention of a sigmoid loop with a whirl sign

**Diagnostic assessment:** biological tests revealed a hemoglobin of 15.5 g/dL and hyperleukocytosis of 21000/L. An unprepared abdominal X-ray showed a dilated sigmoid colon with multiple hydro-aeric levels. Abdominal computed tomography (CT) revealed a twisted and significantly distended dolichosigmoid loop, 11cm in diameter, with a thinning of the intestinal wall and multiple hydro-aeric levels (Figure 1), and concluded a diagnosis of intestinal obstruction with strangulation with possible acute generalized peritonitis.

**Figure 2:**
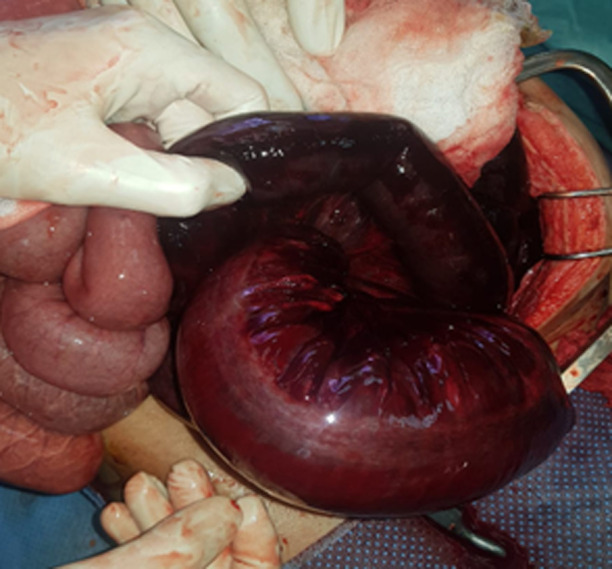
intra-operative picture showing the Ileosigmoid knot with gangrenous ileum and sigmoid colon

**Therapeutic interventions:** after the resuscitation measures, an exploratory laparotomy was indicated. with the exploration; a hemorrhagic effusion of 1.5 liters. The terminal ileum was wound on the base of the sigmoid colon and on its meso in a 360° clockwise circumference, resulting in a 150cm strangulation and necrosis of the ileum from the ileocecal junction, corresponding to a type I ileosigmoid node (Figure 2).

The sigmoid colon was carefully twisted, and the ileal node opened. The 150cm nonviable distal ileum and sigmoid colon were resected. An ileo-ileal anastomosis and a Hartmann colostomy were performed. An abdominal drain was placed in the pelvic cavity after abundant washing with saline. The length of the remaining small intestine measured 160cm. The drainage was removed after 72 hours. The postoperative course was uneventful and the patient was discharged after 8 days. After three months, the patient underwent a successful Hartmann colostomy reversal without complications.

**Follow-up and outcome of interventions:** the patient was advised for regular follow-up.

**Patient perspective:** he said his diagnosis has been explained well and received good support.

**Informed consent:** the patient gave informed consent to publish this case report and any related photos.

### Case 2

**Patient information:** a 60-year-old man with no specific pathological history presented to the emergency department with severe abdominal pain and complete cessation of bowel movements, which had begun 48 hours earlier. The pain was intense and localized in the umbilical region.

**Diagnostic assessment:** physical examination revealed diffuse abdominal distension with diffuse abdominal tenderness to palpation. After initial conditioning, an abdominal X-ray revealed numerous hydro-aeric levels. A computed tomography (CT) scan was performed, revealing twisted and knotted mesenteries of the sigmoid colon and terminal ileum, as well as dilatation of the sigmoid colon and multiple hydro-aeric levels in the intestines. Laboratory tests on admission showed hyperleukocytosis with neutrophilia (white blood cell count 16.4 x 10^3^/L, neutrophils 86%). The diagnosis of acute mechanical intestinal obstruction was established and urgent surgery was indicated.

**Figure 3:**
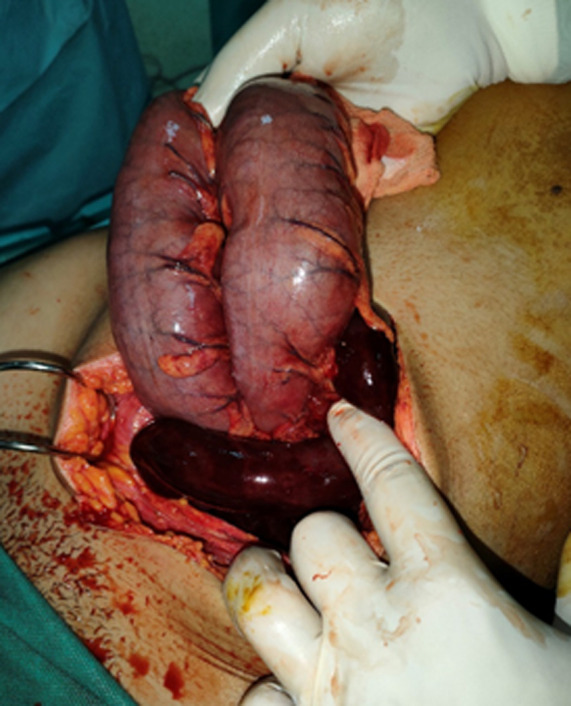
intraoperative image showing ileal knot around the base of the sigmoid

**Figure 4:**
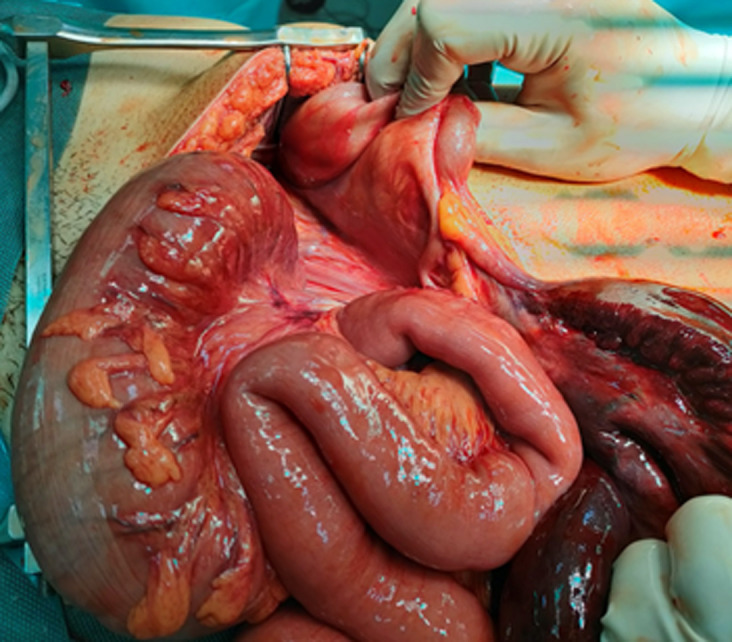
intraoperative image after unwrapping the knot

**Therapeutic interventions:** the patient underwent an exploratory laparotomy. During the operation, necrosis of the last ileal loop was observed, extending from the sigmoid colon to 5cm upstream of the ileocecal junction (Figure 3). The knot was carefully untangled and the sigmoid colon recolored normally, so it was not necrotic (Figure 4). However, one and a half meters of the distal ileum was found to be necrotic and was resected. An ileo-ileal anastomosis was then performed and an abdominal drain was inserted into the pelvic cavity. The patient's postoperative evolution was favorable, and he was discharged from the hospital after five days.

**Follow-up and outcome of interventions:** the patient was advised for regular follow-up.

**Patient perspective:** he said his diagnosis has been explained well and received good support.

**Informed consent:** the patient gave informed consent to publish this case report and any related photos.

### Case 3

**Patient information:** an 83-year-old woman sought emergency care for two days of no bowel movements and gas, accompanied by intense abdominal pain.

**Figure 5:**
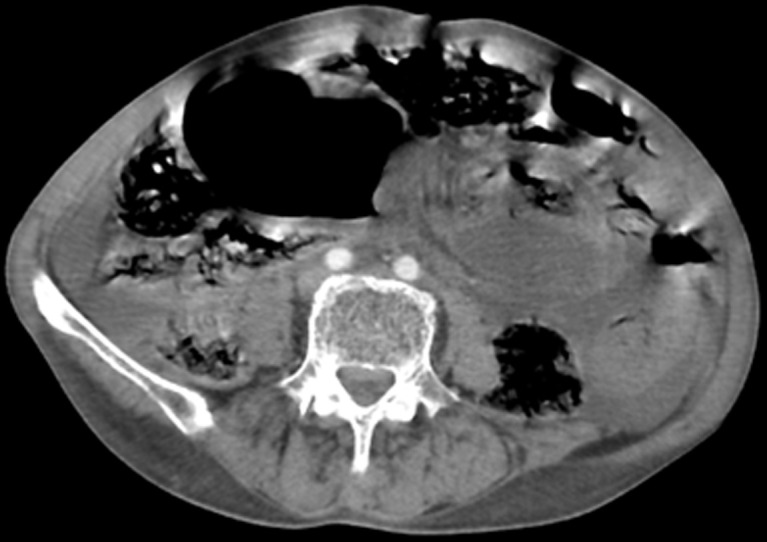
a computed tomography scan showing a radial arrangement of the small bowel in the left lower quadrant with a transition zone, associated with a sign of whirlpool

**Diagnostic assessment:** during the clinical examination, a distended abdomen, abdominal bloating, diffuse mucocutaneous pallor, and an empty rectal ampulla were observed. The blood count showed anemia with a hemoglobin level of 8 g/dl. The CT scan revealed a radial arrangement of the small intestine in the left lower quadrant, with a transition zone and a whirl sign (Figure 5). Secondly, it demonstrated the distension of a sigmoid loop exceeding 70 mm, preceding a beak-like transition zone. There was no dilation of the colon upstream, indicating a sigmoid volvulus. The spontaneously hyperdense appearance of the ileum walls, accompanied by a lack of enhancement, and the presence of peritoneal effusion were consistent with ischemic bowel distress.

These radiological findings suggested a dual obstruction caused by ileal and sigmoid volvulus.

**Figure 6:**
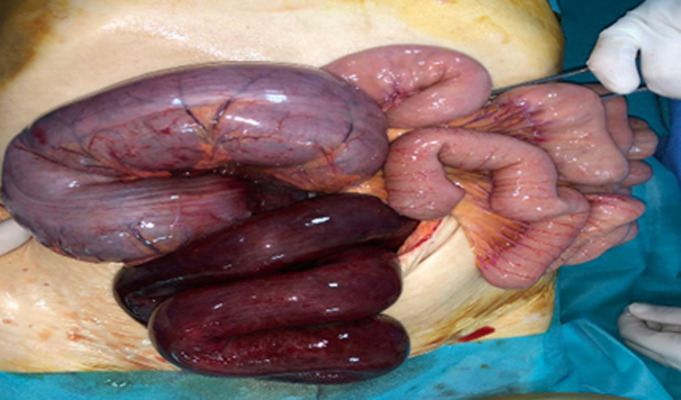
image of an ileosigmoid knot

**Figure 7:**
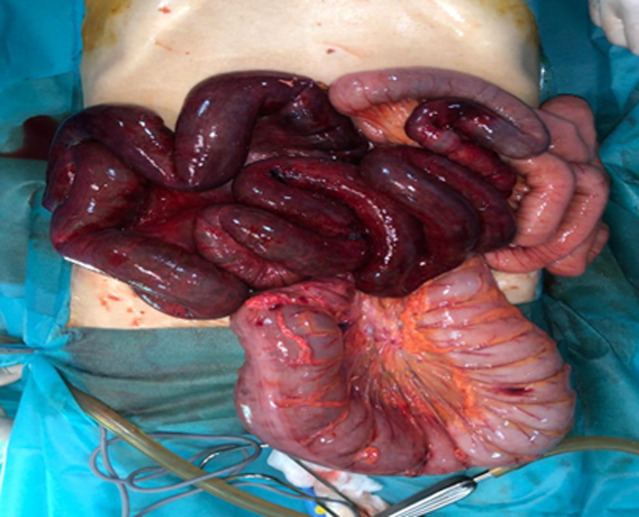
intraoperative view after the knot was unwrapped, sigmoid is viable

**Therapeutic interventions:** the patient underwent emergency surgery, and the volvulus of the ileum caused by torsion of the sigmoid loop was confirmed, forming an ileosigmoid knot. Extensive necrosis of the ileum was observed (Figure 6, Figure 7), leading to ileal resection and intestinal anastomosis.

**Follow-up and outcome of interventions:** the patient was advised for regular follow-up.

**Patient perspective:** she said her diagnosis has been explained well and received good support.

**Informed consent:** the patient gave informed consent to publish this case report and any related photos.

## Discussion

Intestinal knot syndromes occur when a section of the intestine wraps around the base of another bowel loop, leading to potentially serious complications. Among these syndromes, ileosigmoid knotting is the most common, characterized by the winding of the ileum around the base of the sigmoid mesocolon, resulting in rapid gangrene development in both segments [2,3,6]. A number of factors favour the occurrence of ileosigmoid knotting, including a very long, hypermobile small intestinal mesentery, a sigmoid dolichocolon with a narrow meso, and a diet rich in dietary fiber when the small intestine is empty [7].

Pain, abdominal distension, and obstruction form the classic volvulus triad, observed in 27.3% to 100% of patients [8]. In the literature, three types of ileosigmoid knots have been described [3,8,9]: type I: the ileum is the active component and wraps clockwise or anticlockwise around the passive sigmoid colon; type II: the sigmoid colon is the active segment; type III: the entire ileocecal segment wraps around the sigmoid colon.

Type I is the most common type of ileosigmoid knot, followed by type II. The main symptom of this pathological entity is sudden-onset abdominal pain, usually accompanied by vomiting and abdominal distension, which rapidly worsens. The initial presentation may be a hypovolemic shock. Physical examination usually reveals abdominal distension, tenderness, and even generalized defensiveness. Imaging usually shows a double loop of dilated sigmoid umbra and fluid levels in the small intestine. However, CT scans can show specific signs such as the whirl sign caused by twisting of the bowel and sigmoid mesocolon, median deviation of the cecum, and descending colon. But may not be feasible in unstable patients [6,7].

Prompt laparotomy should not be delayed following effective resuscitation. Gangrenous bowel is often encountered during surgery, with viable small and large intestines being a rare finding [3]. Various surgical approaches have been employed to manage ileosigmoid knotting, including [3]: ileum resection + primary anastomosis + sigmoid derotation, or iileum resection + primary anastomosis + sigmoidectomy + sigmoidostomy, ileum resection + end ileostomy + sigmoid resection + primary anastomosis, ileum diversion + sigmoid resection + primary anastomosis, ileum resection + primary anastomosis + sigmoid resection + primary anastomosis and ileum resection + primary anastomosis + sigmoid resection + Hartmann´s procedure/colostomy.

Among these therapeutic options, the most commonly performed procedure is ileal resection with intestinal anastomosis combined with sigmoid resection and the Hartmann procedure. This was the case in one of our patients, while in the other two cases, we opted for resection followed by ileal anastomosis with derotation of the sigmoid. Mortality rates associated with the ileosigmoid node vary from 0 to 48% and depend on factors such as the duration of symptoms, the presence of necrosis, and the patient's general state of health, including the presence of septicemic shock [5].

## Conclusion

Ileosigmoid knotting, although rare, represents a significant cause of acute intestinal obstruction, characterized by the entanglement of the ileum around the base of the redundant sigmoid colon and mesosigmoid. improving the survival rate of patients with acute intestinal occlusion secondary to an ileosigmoid node requires a better understanding of the pathophysiology of this condition, and surgical intervention as soon as diagnosis is made after resuscitation, as well as the most effective surgical procedure based on surgical examination.
